# Effects of Chinese Propolis in Protecting Bovine Mammary Epithelial Cells against Mastitis Pathogens-Induced Cell Damage

**DOI:** 10.1155/2016/8028291

**Published:** 2016-06-28

**Authors:** Kai Wang, Xiao-Lu Jin, Xiao-Ge Shen, Li-Ping Sun, Li-Ming Wu, Jiang-Qin Wei, Maria Cristina Marcucci, Fu-Liang Hu, Jian-Xin Liu

**Affiliations:** ^1^College of Animal Sciences, Zhejiang University, Hangzhou 310058, China; ^2^Institute of Dairy Science, Key Laboratory of Molecular Animal Nutrition, Ministry of Education, Zhejiang University, Hangzhou 310058, China; ^3^Institute of Apicultural Research, Chinese Academy of Agricultural Sciences, Beijing 100093, China; ^4^CSIRO Food and Nutrition, Adelaide, SA 5000, Australia; ^5^Laboratory of Natural Products, Pharmacy Department, University Anhanguera of São Paulo, 1813 São Paulo, SP, Brazil

## Abstract

Chinese propolis (CP), an important hive product, can alleviate inflammatory responses. However, little is known regarding the potential of propolis treatment for mastitis control. To investigate the anti-inflammatory effects of CP on bovine mammary epithelial cells (MAC-T), we used a range of pathogens to induce cellular inflammatory damage. Cell viability was determined and expressions of inflammatory/antioxidant genes were measured. Using a cell-based reporter assay system, we evaluated CP and its primary constituents on the NF-*κ*B and Nrf2-ARE transcription activation. MAC-T cells treated with bacterial endotoxin (lipopolysaccharide, LPS), heat-inactivated* Escherichia coli,* and* Staphylococcus aureus* exhibited significant decreases in cell viability while TNF-*α* and lipoteichoic acid (LTA) did not. Pretreatment with CP prevented losses in cell viability associated with the addition of killed bacteria or bacterial endotoxins. There were also corresponding decreases in expressions of proinflammatory IL-6 and TNF-*α* mRNA. Compared with the mastitis challenged cells, enhanced expressions of antioxidant genes HO-1, Txnrd-1, and GCLM were observed in CP-treated cells. CP and its polyphenolic active components (primarily caffeic acid phenethyl ester and quercetin) had strong inhibitive effects against NF-*κ*B activation and increased the transcriptional activity of Nrf2-ARE. These findings suggest that propolis may be valuable in the control of bovine mastitis.

## 1. Introduction

Inflammation of the mammary gland (mastitis) caused by invading pathogens is common among lactating dairy cows and leads to considerable economic loss through reduced milk yield, impaired milk quality, and increased veterinary costs [1]. Globally, mastitis is one of the most costly bovine diseases for the dairy industry, estimated to cost approximately $1.8 billion annually in the United States [2].

Mastitis is primarily categorized as subclinical or clinical mastitis. The differences in these types of mastitis are based on the invading pathogen(s) and resolution of the infection. Clinical mastitis is stronger and more acute than the subclinical mastitis, and it is caused mainly by Gram-negative bacteria such as* Escherichia coli*. The subclinical mastitis elicits milder inflammation and persists over the life span of infected cows. Gram-positive bacteria, like* Staphylococcus aureus*, are involved with the subclinical mastitis infection. Endotoxin (lipopolysaccharide, LPS) and lipoteichoic acid (LTA) are considered as the main virulence factor of* E. coli* and* S. aureus*, respectively [3, 4]. Moreover, proinflammatory cytokine tumor necrosis factor- (TNF-) *α* elicits both local and systemic inflammatory reactions and triggers the inflammatory cascade “acute phase response” in inflammation and participated in the acute phase of coliform mastitis [5, 6].

To suppress proliferation of invading pathogenic mastitis-causing bacteria, modern dairy practice employs several strategies, including teat disinfection, antibiotic therapy, and culling of persistently infected cows [7]. Despite the great effectiveness of antibiotics, their use is coming under increasing public scrutiny due to the possible development of resistant pathogens (like methicillin-resistant* S. aureus*, MRSA) and food safety concerns (like antibiotic residues in milk) [8]. Considering the risks associated with antibiotic therapies for bovine mastitis, development of alternative treatment strategies for management of clinical and subclinical mastitis are warranted.

Propolis is a resinous substance collected by honeybees (*Apis mellifera* L.) from various polyphenol-rich plants [9]. It has been used widely in folk medicine since ancient times and has attracted much attention in recent years for its various biological properties [10]. In our recent studies, we determined that propolis had potent anti-inflammatory effects in macrophages and boosted cellular antioxidant defence systems [11, 12]. Previous literature has shown that propolis could inhibit the growth of several different bacterial strains known to cause mastitis, as well as some antibiotic-resistant* S. aureus* strains [13–15]. Nevertheless, little is known about the effects of propolis on mastitis responses in bovine mammary epithelial cells (bMECs).

In the present study, we studied the impact of the effect propolis when bovine mammary epithelial cells were challenged with heat-killed mastitis-causing bacterial cells, as well as selected agents also associated with tissue response to mastitis. Several isolated compounds from propolis were investigated to clarify the mechanism of action.

## 2. Materials and Methods

### 2.1. Reagents

LPS (*Escherichia coli* 0111:B4), LTA (from* Staphylococcus aureus*), caffeic acid, caffeic acid phenethyl ester (CAPE), chrysin, ferulic acid, galangin, kaempferol, pinocembrin, and quercetin were purchased from Sigma (St. Louis, MO, USA). High performance liquid chromatography- (HPLC-) grade methanol was obtained from Merck (Darmstadt, Germany). Recombinant human TNF-*α* was purchased from Peprotech (Rocky Hill, NJ, USA). Culture plates were obtained from Coring Life Science (Lowell, CA, USA.). The PI/RNase Staining Buffer kit, FITC-conjugated annexin V, and Binding Buffer were obtained from BD Biosciences (San Diego, CA, USA). Other chemicals were of analytical grade and purchased from Sangon Biotechnology (Shanghai, China).

### 2.2. Preparation and Chemical Analysis on Chinese Propolis Extract

Chinese propolis (CP) was obtained from colonies of honeybees,* A. mellifera L.*, in Shandong province in the summer of 2010, and the main plant origin was poplar (*Populus* spp.). The propolis extracts were obtained previously [11]. Briefly, raw propolis (100 g) was extracted by 95% (V/V) ethanol (1 L) and sonicated at 40°C for 3 h. The supernatant was collected and filtered to remove the residues. The raw propolis was extracted for three times. Then the supernatants were collected and evaporated in a rotary evaporator under a reduced pressure at 50°C to evaporate the ethanol. Dried PPE were stored at −20°C until further use. For the* in vitro* studies, CP was redissolved directly in ethanol to a concentration of 20 mg/mL and sterilized using a 0.22 *μ*m syringe filter (Pall, Port Washington, NY, USA). The final concentration of ethanol in the cell culture was less than 0.5% (V/V). Major polyphenolic compounds in CP were determined by HPLC as described previously [16].

### 2.3. Cell Culture and Mastitis Pathogen Challenges

Bovine MEC line MAC-T cells were generously provided by Professor Fengqi Zhao (University of Vermont, Burlington, USA) which were cultured in high-glucose Dulbecco's modified Eagle's medium (DMEM, Hyclone, Fremont, CA, USA) supplemented with 100 U/mL of penicillin, 100 *μ*g/mL streptomycin, and 10% (V/V) heat-inactivated fetal bovine serum (FBS, Gibco, Carlsbad, CA, USA) at 37°C and 5% CO_2_ in a humidified incubator. We challenged these cells with heat-inactivated bacteria particles of the mastitis-causing pathogens* E. coli* strain 1303 [17] and* S. aureus* Newbould 305 [18]. Details regarding the culture of* E. coli* or* S. aureus* pathogens and usages of these heat-inactivated bacteria particles to challenge bMECs were described previously [4].* E. coli* and* S. aureus* strains were grown (37°C) in Lysogeny broth (LB) medium to the logarithmic phase of culture growth. After that, plating of dilution series was used to calibrate cell counts. Heat inactivation was performed in an 80°C water bath for 1 h to kill all live cells and verified through control plating. Subsequently, cells were spun down, washed twice with PBS, and later then resuspended with DMEM at a density of 5 × 10^8^ cells/mL. Aliquots were stored frozen at −20°C until used.

### 2.4. Cell Viability Assay

Cell viability assay was performed using the CCK-8 kit (Dojido, Kumamoto, Japan) according to the manufacture's instruction. Briefly, 10 × 10^4^/mL MAC-T cells were seeded into 96-well culture plates. After 24 h incubation, cells in each well with specific treatment were incubated with 10 *μ*L of CCK-8 at 37°C for 2 h before measuring the OD at 450 nm with a microplate reader (SpectraMax M5, Molecular Devices, Sunnvale, CA, USA). Cell viability was also confirmed by trypan blue exclusion.

### 2.5. Annexin V/PI Assays for Cell Apoptosis

Cells were stained with annexin V-FITC and PI and then evaluated for apoptosis by flow cytometry according to the manufacturer's protocol (BD Biosciences, San Diego, CA, USA). Briefly, 10^6^ cells were washed twice with PBS and stained with 5 *μ*L of annexin V and 5 *μ*L of PI (50 *μ*g/mL) in washing buffer from the kits for 15 min at room temperature in the dark. Apoptotic cells that were identified using a BD FACSCalibur flow cytometer. Apoptotic cells were counted in the lower right quadrant corresponding to early apoptotic cells (annexin V-positive) and those in the upper right quadrant corresponded to late apoptotic cells (annexin V- and PI-positive).

### 2.6. Quantitative Real-Time Polymerase Chain Reaction (PCR)

Total RNA was extracted with the RNA pure kit (Aidlab Biotechnologies Co., Ltd., Beijing, China) following the manufacturer's instructions. Reverse transcription was done using the PrimeScript RT reagent kit (TaKaRa, Dalian, China) with 1 *μ*g total RNA. Quantitative real-time PCR was carried out with SYBR premix EX Taq (TaKaRa) following the manufacturer's instructions with 1 : 10 diluted cDNA template and using a standard two-step reaction [19]. Expression of the housekeeping gene *β*-actin was used for normalization. Primers for target genes that covered introns were designed with the Primer5 software (Premier Biosoft International, Palo Alto, CA) and listed in Table 1.

### 2.7. Cell Transfection and Luciferase Assay

Reporter assays are performed according to our previous methods [19]. After overnight incubation, 30 ng of firefly luciferase reporter plasmid pGL4.2-NF-*κ*B-Luc and pGL4.37-luc2P/ARE/Hygro vector (Promega, Madison, WI, USA) were transfected to drive transcription of NF-*κ*B and ARE, respectively, by using Lipofectamine 2000 (Invitrogen, Carlsbad, CA, USA). Sea pansy luciferase reporter plasmid (pRL-TK, 5 ng) was transfected to normalize the transfection efficiency. The pcDNA3.1 empty vector was used to compensate the total expression plasmids to 500 ng/well. Luciferase activities were measured 24 h after specific treatments by using the Dual-Glo Luciferase Assay System (Promega).

### 2.8. Statistical Analysis

Data are expressed as the means ± SD from at least three independently performed experiments. One-way analysis of variance (ANOVA) followed by Student-Newman-Keuls (SNK) multiple-comparison test was used to determine statistical significance for multiple comparisons, and Student's *t*-test was used for comparisons of two groups. Statistical significance was defined at ^*∗*^ *P* <  0.05. All statistical tests were carried out using SPSS 17.0.

## 3. Results

### 3.1. Chemical Composition of Chinese Propolis

We analyzed the major polyphenolic compounds in Chinese propolis using our previously developed HPLC method [16]. Their relative concentrations in CP are listed in Table 2. The major polyphenolic components were chrysin, pinocembrin, pinobanksin, galangin, and CAPE.

### 3.2. Effects of Chinese Propolis on Mastitis Pathogens-Induced Cell Viability Decreases and Cell Apoptosis in MAC-T Cells

**Figure 1:**
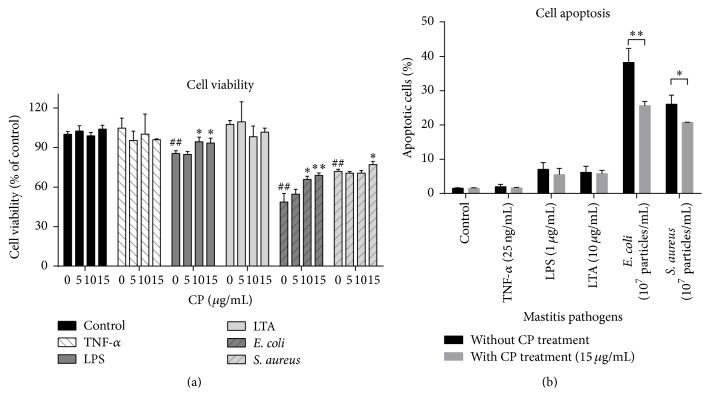
Effects of propolis on mastitis pathogen-induced cell viability decreases and cell apoptosis in MAC-T cells. (a) MAC-T cells were treated with propolis and/or various mastitis pathogens, including proinflammatory cytokine (TNF-*α* 25 ng/mL), bacterial cell wall components (LPS, 1 *μ*g/mL; LTA, 10 *μ*g/mL), and heat-killed mastitis strains (*Escherichia coli* and* Staphylococcus aureus*, 107 particles/mL) with indicated concentrations of propolis for 24 h. The CCK-8 assay was performed to determine cell viability. ^##^ *P* < 0.01 and ^###^ *P* < 0.001 significantly different from untreated cells. ^*∗*^ *P* < 0.05 and ^*∗∗*^ *P* < 0.01 significantly different from mastitis pathogens-treated cells. (b) After being pretreated with or without Chinese propolis (15 *μ*g/mL) for 1 h, MAC-T cells were challenged with various mastitis pathogens for 24 h. Cell apoptosis was analyzed by flow cytometry analysis using annexin V-FITC and PI staining. The data are expressed as the mean ± SD (*n* = 3). ^*∗*^ *P* < 0.05 and ^*∗∗*^ *P* < 0.01.

As shown in Figure 1(a), not all of these stimuli caused cell viability decreases in MAC-T cells. Only LPS and heat-killed* E. coli* and* S. aureus*, but not TNF-*α* and LTA stimulation, lead to significant cell viability losses (15% to 52%, *P* = 0.0009, 0.0041, and 0.001 for LPS,* E. coli,* and* S. aureus*, resp.). Heat-killed bacterial strains caused more serious cell viability losses than LPS.

To test the effects of CP on protecting against the cell viability decreases caused by mastitis pathogens, various concentrations of CP were added to MAC-T cells with or without added bacterial cells. Also shown in Figure 1(a), tested concentrations of CP (5, 10, and 15 *μ*g/mL) pretreatment were safe to MAC-T cells. CP pretreatment (10 and 15 *μ*g/mL) significantly mitigated cell viability loss by LPS (*P* < 0.05) and heat-killed* E. coli* (*P* < 0.05).* S. aureus*-induced cell viability losses can be inhibited by 15 *μ*g/mL CP pretreatment (*P* < 0.05).

Cell apoptosis analysis by flow cytometry showed that 10^7^ particles/mL heat-killed mastitis strains caused a substantially increased percentage of apoptotic cells, but none of the other stimuli, TNF-*α*, LPS, or LTA, cause any changes on apoptotic cell numbers (Figure 1(b)). Nevertheless, pretreatment with 15 *μ*g/mL CP in MAC-T cells showed a significantly lower number of apoptotic cells (*P* < 0.05) compared to heat-killed* E. coli*- and* S. aureus*-treated cells, which was consistent with the above result obtained from CCK-8 cell viability assay.

### 3.3. Effects of CP Treatment on Proinflammatory Responses following Mastitis Pathogen Challenges

**Figure 2:**
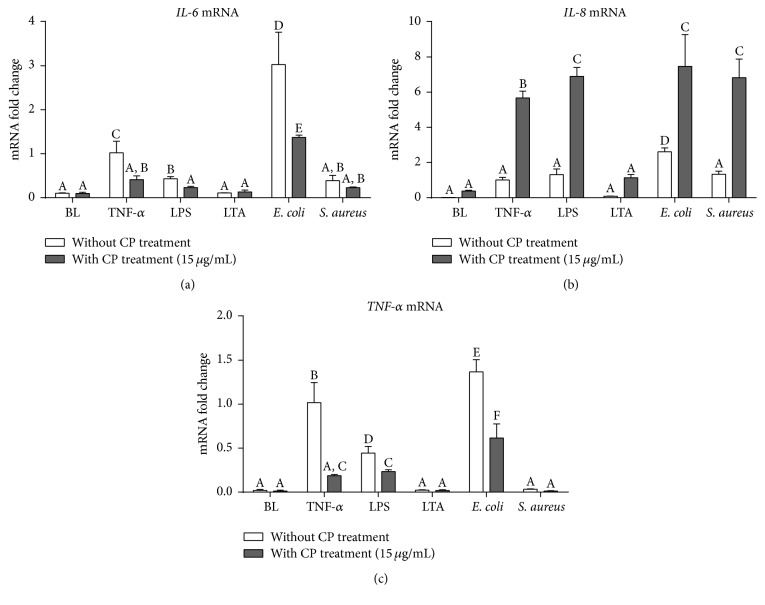
Effects of CP treatment on proinflammatory responses following mastitis pathogen challenges in MAC-T cells. Quantitative PCR analysis of inflammatory cytokine genes, IL-6 (a), IL-8 (b), and TNF-*α* (c), showing gene expressions after 6 h incubation of MAC-T cells with each different mastitis pathogen, including proinflammatory cytokine (TNF-*α* 25 ng/mL), bacterial cell wall components (LPS, 1 *μ*g/mL; LTA, 10 *μ*g/mL), and heat-killed mastitis strains (*E. coli* and* S. aureus*, 10^7^ particles/mL). Ct values of target genes were normalized to the value of *β*-actin and relative gene expressions in TNF-*α* control group were arbitrarily set to one. The data are shown as mean ± SD from three independent experiments and were analyzed by one-way ANOVA with the Student-Newman-Keuls method. The means with different superscripts are significantly different (*P* < 0.05).

As shown in Figure 2, all of these mastitis pathogens, except for LTA, lead to significant increases of IL-6 and IL-8 (*P* < 0.05). LTA and heat-killed* S. aureus* stimulation also failed to induce expression of TNF-*α* compared with uninfected cells (*P* > 0.05). On the contrary, heat-killed* E. coli* lead to the strongest inductive effects compared to other mastitis stimuli. CP-pretreated MAC-T cells have much slighter changes of IL-6 and TNF-*α* mRNA compared to their corresponding stimuli controls. Surprisingly, CP lead to ~8-fold changes of IL-8, significantly higher than TNF-*α*, LPS, and heat-killed* E. coli* stimuli (*P* < 0.05).

### 3.4. Effects of CP Treatment on Cellular Antioxidant Defense Gene Expression following Mastitis Pathogen Challenges

**Figure 3:**
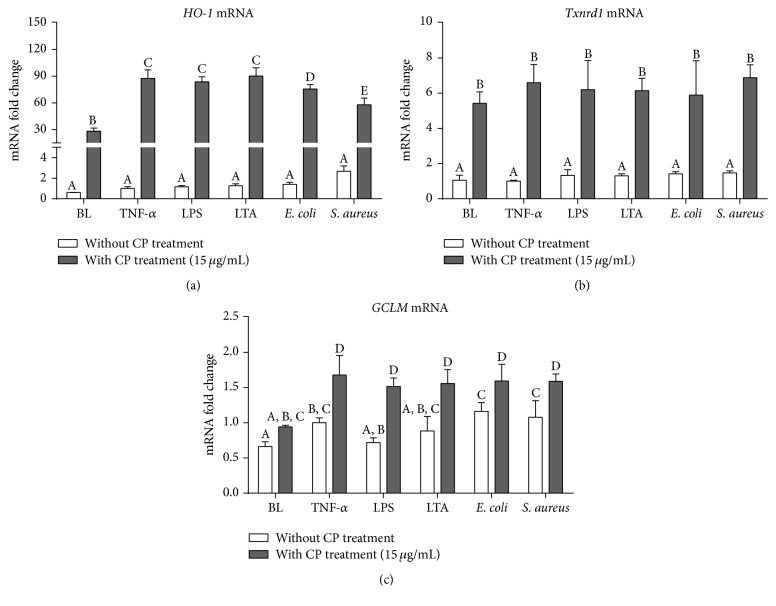
Effects of CP treatment on cellular antioxidant defense gene expressions following mastitis pathogen challenges in MAC-T cells. Quantitative PCR analysis of cellular antioxidant defense genes, HO-1 (a), Txnrd-1 (b), and GCLM (c), showing gene expressions after 6 h incubation of MAC-T cells with each different mastitis pathogen, including proinflammatory cytokine (TNF-*α* 25 ng/mL), bacterial cell wall components (LPS, 1 *μ*g/mL; LTA, 10 *μ*g/mL), and heat-killed mastitis strains (*E. coli* and* S. aureus*, 10^7^ particles/mL). Ct values of target genes were normalized to the value of *β*-actin and relative gene expressions in TNF-*α* control group were arbitrarily set to one. The data are shown as mean ± SD from three independent experiments and were analyzed by one-way ANOVA with the Student-Newman-Keuls method. The means with different superscripts are significantly different (*P* < 0.05).

As shown in Figure 3(c), only GCLM expression was affected by several mastitis pathogens (TNF-*α* and two heat-killed mastitis strain particles). We noticed that CP treatment in MAC-T cells leads to substantial increases in HO-1, Txnrd-1, and GCLM expressions (*P* < 0.05). HO-1 expressions were further elevated in CP-pretreated MAC-T cells undergoing mastitis challenges (Figure 3(a)). Upregulated Txnrd-1 was unchanged in CP-treated MAC-T cells (Figure 3(b), *P* < 0.05), regardless of these different mastitis pathogens. Furthermore, CP treatment itself cannot induce expression of GCLM but it substantially increased in mastitis pathogen challenged cells along with CP. These results indicated that CP could elicit the antioxidant defense system in the bMECs cells undergoing mastitis challenges.

### 3.5. Time and Dose-Response Effects by CP Treatment on Proinflammatory Cytokines and Cellular Antioxidant Gene Expressions in TNF-*α* Stimulated MAC-T Cells

**Figure 4:**
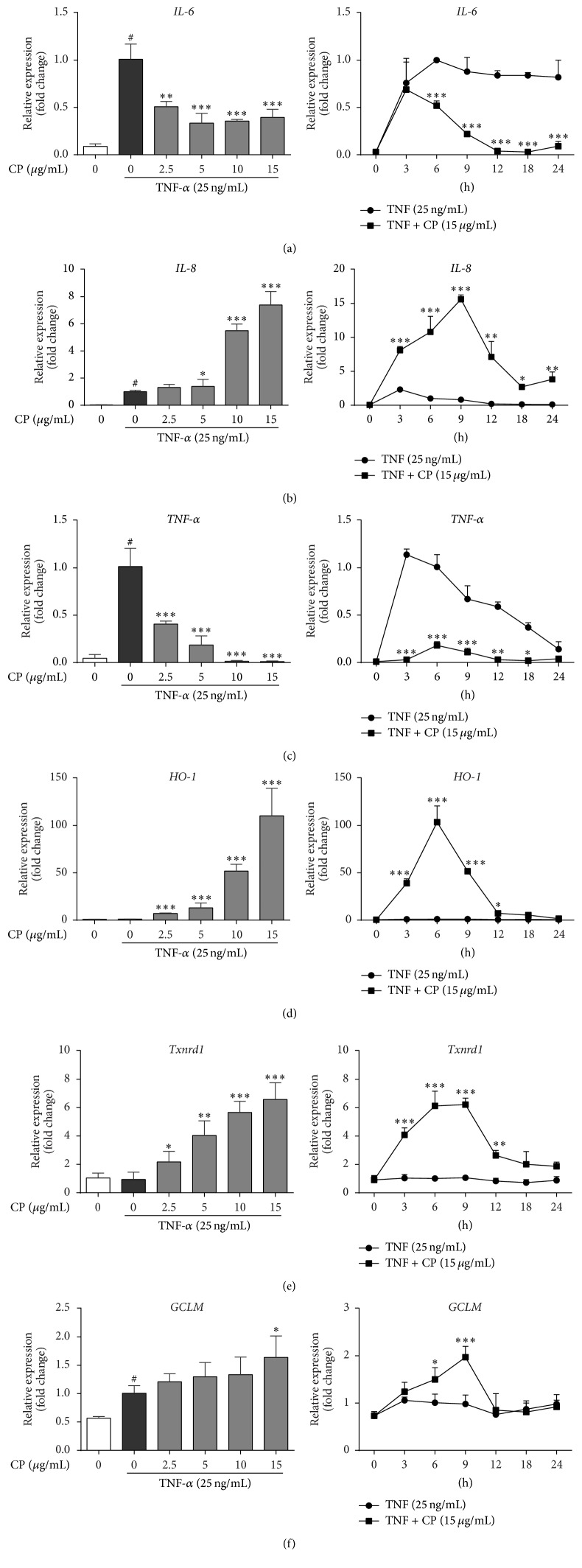
Time and dose effects of CP treatment on inflammatory cytokine and cellular antioxidant defense gene expressions in TNF-*α* stimulated MAC-T cells. MAC-T cells were preincubated with different concentrations of propolis for 1 h and then stimulated with 25 ng/mL TNF-*α* for various time periods (time effects, right) or preincubated with various concentrations of propolis (dose effect, left) and then stimulated with 25 ng/mL TNF-*α* for 6 h. IL-6 (a), IL-8 (b), TNF-*α* (c), HO-1 (d), Txnrd-1 (e), and GCLM (f) mRNA expressions were quantified by real-time PCR. Ct values of target genes were normalized to the value of *β*-actin. Relative gene expression in TNF-*α* stimulation for 6 h was arbitrarily set to one. The data are shown as mean ± SD from three independent experiments. ^#^ *P* < 0.05 compared to the normal control and ^*∗*^significantly different from the TNF-*α* treated control (*P* < 0.05). ^*∗*^ *P* < 0.05, ^*∗∗*^ *P* < 0.01, and ^*∗∗∗*^ *P* < 0.001.

As shown in Figure 4, the increased expressions of proinflammatory cytokines IL-6 (Figure 4(a)) and TNF-*α* (Figure 4(c)) were suppressed by CP in a time- and dose-dependent manner. As for another important chemokine, IL-8, its expression was significantly driven by TNF-*α* and immediately peaked at 3 h after TNF stimulus (Figure 4(b)). CP treatment strongly promoted IL-8 expression, which reached its peak after 9 h TNF stimulus. We also found that TNF-*α* had very limited effects on antioxidant gene expressions, HO-1, TXRND, and GCLM (Figures 4(d)–4(f)).

### 3.6. CP and Its Active Molecules Suppressed NF-*κ*B and Increased Nrf2-ARE Transcriptional Activity

**Figure 5:**
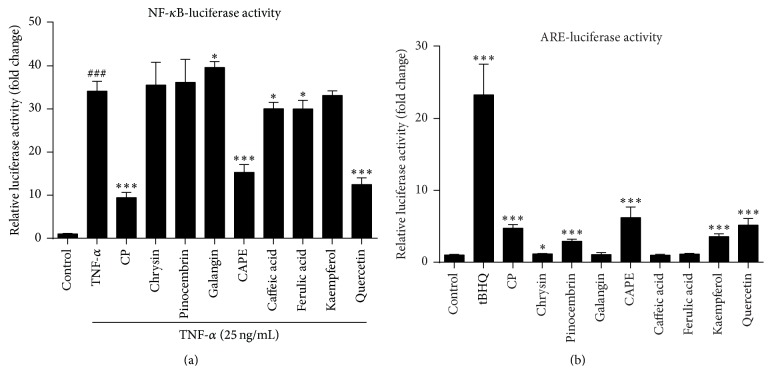
Chinese propolis and its major bioactive compounds suppressed mastitis pathogen-induced NF-*κ*B activation and induces ARE transcriptional activity. (a) Effects of CP on mastitis pathogen-induced NF-*κ*B promoter activation in HEK-293T cells. Cells were pretreated with 20 *μ*g/mL CP and identified isolated active compounds from propolis (caffeic acid, 50 *μ*M; CAPE, 5 *μ*M; chrysin, 50 *μ*M; ferulic acid, 50 *μ*M; galangin, 25 *μ*M; kaempferol, 50 *μ*M; pinocembrin, 50 *μ*M; and quercetin, 50 *μ*M) for 1 h and then stimulated with TNF-*α* (25 ng/mL) for another 12 h. ^###^ *P* < 0.001 compared to the vehicle control; ^*∗*^ *P* < 0.05 and ^*∗∗∗*^ *P* < 0.001 compared to TNF-*α* control. (b) Effects of propolis on mastitis pathogens-induced ARE promoter activation in HEK-293T cells. Cells were treated with CP or identified isolated active compounds. tBHQ (15 *μ*M) treatment for 7 h was used as ARE positive control. ^*∗*^ *P* < 0.05 and ^*∗∗∗*^ *P* < 0.001 compared to the vehicle control. The data represent the mean ± SD of four independent experiments.

To determine the effects of CP as well as its purified compounds on NF-*κ*B and ARE transcriptional activity, luciferase reporter assays were applied in HEK-293T cells. As shown in Figure 5, NF-*κ*B activity was significantly decreased by approximately 70% (*P* < 0.001). The luciferase activity derived from the ARE promoter was consistently increased by approximately 4.7-fold after treating with 20 *μ*g/mL CP (Figure 5(b), *P* < 0.001). Moreover, significant decreases in NF-*κ*B activity were found in cells treated with CAPE (5 *μ*M, 45%, *P* < 0.001), quercetin (50 *μ*M, 73%, *P* < 0.001) caffeic acid (50 *μ*M, 12%, *P* < 0.05), and ferulic acid (50 *μ*M, 11%, *P* < 0.05) (Figure 5(a)). In parallel, the luciferase activity derived from the ARE promoter was consistently increased after treatment with CAPE (5 *μ*M, 2.5-fold, *P* < 0.001), quercetin (50 *μ*M, 5.1-fold, *P* < 0.01), kaempferol (50 *μ*M, 3.6-fold, *P* < 0.001), and pinocembrin (50 *μ*M, 2.9-fold, *P* < 0.01).

## 4. Discussion

Bovine mastitis is usually caused by bacteria and treated with antibiotics. Regardless of increased pressures of cutting down the antibiotic usage in the husbandry in the European Union and US, mastitis treatment is continued after the initial usage of antibiotics, mainly for chronically occurring subclinical mastitis [20]. The increasing scientific data on the benefit of natural products encourages us to explore novel nonantibiotic agents for mastitis therapies. In this study, the potential utilization of Chinese propolis as an anti-inflammatory reagent and immune modulator against bovine mastitis was examined in bMECs.

Previous studies have demonstrated that propolis has great antimicrobial properties against bacterial fungi, protozoan, and even yeast pathogens [21]. Despite chemical variations of propolis from different geographic origins/plant sources, the antibacterial activity by propolis always exists. In accordance with published literature, Chinese propolis extracts on Gram-positive* S. aureus* cells appear to be bactericidal but show only limited activity against Gram-negative* E. coli* bacteria [21]. Based on the literature, we applied 10^7^ particles/mL of the heat-inactivated bacteria to the bMECs, which is approximately 30 particles challenged per host cell [22, 23]. This approach represented a reliable robust stimulus model considering that 10^4^ to 10^6^ CFU of bacteria particles per mL as peak values in the milk were produced from cows after mastitis infection [13]. Also, we confirmed that propolis' bactericidal effects are potent in controlling mastitis-causing* S. aureus* strains [15], which are most frequent but more difficult to eradicate by conventional antibiotic therapies [24].

It has been known that acute coliform mastitis is caused by endotoxin-producing acute coliform bacteria (specifically, e.g.,* E. coli*,* Enterobacter*, and* Klebsiella* spp.) and is characterized by a rapid and intense increase in the somatic cell count (SCC), while subclinical mastitis caused by* S. aureus* is characterized by a more moderate and delayed SCC increase that leads to chronic and, in some circumstances, life-long infection. Mammary tissue damages induced by mastitis pathogens also have been shown to be linked with increased SCC and mainly induced by either apoptotic or necrotic mammary cells [25]. We explored and compared the effects of major different various mastitis pathogens on bMECs cell viability and cell apoptotic features. We found that TNF-*α* and LTA stimulation did not cause any significant losses on the cell viability or cell apoptosis to bMECs, coinciding with other* in vitro* studies using bovine mammary cells [1, 5]. Meanwhile, previous findings on proapoptotic effects by heat-killed* E. coli* and* S. aureus* were inhibited by CP pretreatment, suggesting that CP has modulating effects on the cellular apoptosis cascade, such as blocking the activation of caspases [1, 26]. We noticed that the* E. coli* cell wall component LPS only decreased cell viability but did not induce cell apoptosis, suggesting that the influences of bacterial cell wall components on bMECs may be different from those mastitis bacteria strains [4]. Moreover, as the internalization process of bacteria pathogens to the cells differs among bacteria strains, the different signal transduction mechanisms requiring the interaction between CP and cell wall components as well as the host cells are in need to be further clarified [27].

Bovine mastitis is initiated by the entry of bacteria through the teat canal. Shortly after entry of the invading pathogen, the resident leukocytes together with epithelial cells initiate the inflammatory response necessary to eliminate the invading bacteria. Different mediators of inflammation, especially some inflammatory cytokines, which are expressed during this stage will participate in the innate immune defense to respond to the earliest stages of infection after mastitis pathogen/stimulus exposure. IL-6 and TNF-*α* are the two most predominant proinflammatory cytokines during the acute phase responses of mastitis. In heat-inactivated* E. coli* challenged bMECs, significant increases of IL-6 and TNF-*α* gene expression were observed (Figure 2), a finding consistent with a previous study using a different mastitis strain of* E. coli* [4]. Compared to LPS and TNF-*α*, heat-inactivated* E. coli* elicited stronger inductive effects on these inflammatory cytokines expressions, suggesting that some other virulence factors from* E. coli* (like lipoproteins or flagellum) may be involved in the innate immune defense of the bovine epithelium cells [23, 28]. In contrast to* E. coli* challenged models, lower levels of inflammatory response were found after* S. aureus* compared with* E. coli*, which has been found by previous studies. In these studies no increases in the concentrations of TNF-*α*, IL-1*β*, and IL-8 in milk were found after intramammary challenge by* S. aureus* [29]. In our experiments, we did not notice any significant changes on these inflammatory cytokine genes following* S. aureus* or LTA stimulation. Previous reports have suggested that* S. aureus* mastitis is characterized by a more moderate and delayed inflammatory responses and limited cytokine responses [22, 25]. Expanded time-course studies of CP effects by* S. aureus* and LTA stimulation need to be conducted.

Propolis has been used historically as a folk medicine since ancient ages times and has since been safely used as a substitute medicine or supplement to improve human health in modern days [30]. Propolis exerted versatile biological activities, especially for anti-inflammatory effects. Although the effective components in propolis vary significantly according to the mother plant that the honeybees collect it from, the bioactivities of propolis are always present. Our previous studies have confirmed the anti-inflammatory effects of Chinese propolis (poplar type) by protecting murine macrophages from LPS challenges and controlling proinflammatory cytokine releases in LPS-induced mice endotoxemia [19]. Similar to our previously published results, the proinflammatory cytokine expressions (IL-6 and TNF-*α*) were inhibited by CP treatment following coliform mastitis pathogen challenges (heat-inactivated* E. coli* as well as endotoxin). Besides, through our comprehensive TNF-*α* stimulation model, we found that CP effectively inhibits the expression of these two genes in a time- and dose-dependent manner. Through our previous studies using LPS challenged murine macrophages models, the inhibition on NF-*κ*B activation by CP may be the source of its anti-inflammatory effects. We knew that polyphenolic compounds are among the most prominent components of propolis because they are considered responsible for most of its properties. In parallel with previous studies, CAPE [31], quercetin [32], and caffeic acid [33] may contribute to less pronounced NF-*κ*B activity following TNF-*α* stimulation.

It should be noted that in this study, the expression of IL-8 (also known as CXCL8) increased in CP-treated cells in several different stimulus models. Various animal models of inflammation have demonstrated IL-8 as a principal chemotactic factor directing neutrophil recruitment to activation at the inflammatory focus. Expression of IL-8 is known to be controlled by multiple transcriptional factors (activator protein, AP, -1, and NF-*κ*B and NF-*κ*B repressing factor, NRF) and involved with some coactivator CBP/300 complexes [34]. It also has been shown that the pivotal importance of C/EBP*β* to activate IL-8 expression in MECs is independent of NF-*κ*B activation [35]. Therefore, in this study, as we have found that the NF-*κ*B transcriptional activity was inhibited by CP and its bioactive compounds, we assumed that other transcriptional factors, like C/EBP*β*, which are possibly activated by CP, may contribute to the enhancement of IL-8 expression in mastitis pathogens challenged MEC.

It is well-known that multiple antioxidant genes may be induced by diverse plant-derived natural products. These antioxidant genes, such as HO-1, Txnrd1, GCLM, SOD, and NQO-1, encode several detoxifying (phase II) enzymes and endogenous antioxidants to provide protections against external stresses, including oxidative stress and inflammation. These major endogenous antioxidants are regulated by various transcription factors, including Nrf2. In particular, Nrf2 has been shown to be involved in the inductions of phase II detoxifying enzymes and antioxidants by natural polyphenols in bMECs suffered from oxidative stress. HO-1 is well-known to be a major antioxidant enzyme that plays an important role in the antioxidant defense against inflammatory stresses in different cell types, such as macrophages [36], keratinocytes [37], and epithelial cells [38, 39]. Thioredoxin reductase (TrxR), which catalyzes the reduction of the active site disulfide of thioredoxin (Trx), is an NADPH-dependent homodimeric oxidoreductase. As TrxR is an antioxidant enzyme that can scavenge ROS, CP-induced TrxR1 expression may indicate the activation of cellular defense systems against TNF and other mastitis pathogen challenges. In addition, reduced glutathione (GSH) can lower the ROS scavenge efficiency. Our recent study found that endogenous antioxidant defenses systems could be activated by Chinese propolis in murine macrophages [12]. In parallel with this study, CP strongly unregulated the HO-1, Txnrd1, and GCLM expressions in bMECs. These increased antioxidant gene expressions are in keeping with CP's inductive effects of Nrf2-ARE transcription activity. Furthermore, several specific active polyphenolic compounds from propolis have been shown to activate Nrf2-ARE signaling, including CAPE [40], kaempferol, quercetin [41], and pinocembrin [42].

## 5. Conclusions

Our studies have demonstrated that Chinese propolis has strong protective effects against various mastitis pathogen insults to bMECs. Apart from CP's bactericidal effects, it clearly prevented cell viability losses/apoptosis induced by LPS and heat-inactivated mastitis strains (*E. *coli and* S. aureus*) in bMECs. These effects were partly achieved by modulating expression of inflammatory cytokine genes and boosting antioxidant defense genes. The possible mechanisms for CP against bovine mastitis may be attributed to its abundant polyphenolic components (mainly CAPE and quercetin), which strongly inhibited NF-*κ*B transcription activity and increased the transcriptional activity of the Nrf2-ARE pathway. Field studies are needed to confirm that propolis may be developed as nonantibiotic therapy strategy for the control of cow mastitis.

## Figures and Tables

**Table 1:** Sequences of primers used for quantitative real-time RT-PCR.

Gene	Primer sequence^a^		Product size (bp)	GenBank accession number
*IL-6*	F	5′-AAACGAGTGGGTAAAGAACGC-3′	143	NM_173923.2
	R	5′-GACCAGAGGAGGGAATGCC-3′		

*IL-8*	F	5′-TCTCAGCCATCTTTTTACCTCAC-3′	176	NM_173925.2
	R	5′-ATCAACATTCTTTCCCATTTCTC-3′		

*TNF-α*	F	5′-CGGTGGTGGGACTCGTA-3′	185	NM_173966.3
	R	5′-AATGCGGCTGATGGTGT-3′		

*HO-1*	F	5′-GGCAGCAAGGTGCAAGA-3′	221	NM_001014912.1
	R	5′-GAAGGAAGCCAGCCAAGAG-3′		

*Txnrd1*	F	5′-GTGTTCACGACTCTGTCGGT-3′	240	NM_174625.3
	R	5′-CTGCCTTCCACGAATCACCT-3′		

*GCLM*	F	5′-GACAAAACCCAGTTGGAGCA-3′	235	NM_001038143.1
	R	5′-AGTACCGCAGTAGCCACAGAG-3′		

*β-actin*	F	5′-CAAGGACCTCTACGCCAAC-3′	257	NM_173979.3
	R	5′-AGAAGCATTTGCGGTGGAC-3′		

^a^F = forward; R = reverse.

**Table 2:** Major phenolic acids and flavonoids presented in Chinese propolis.

Peak number	Compounds	Retention time (min)	Contents (mg/g of extract)
1	Caffeic acid	11.1	4.1 ± 0.3^a^
2	p-Coumaric acid	16.5	1.8 ± 0.7
3	Ferulic acid	19.0	1.2 ± 0.3
4	Isoferulic acid	21.1	1.44 ± 0.0
5	3,4-Dimethoxycinnamic acid	28.5	3.6 ± 0.2
6	Cinnamic acid	31.0	1.4 ± 0.0
7	Pinobanksin	36.2	9.0 ± 0.5
8	Naringenin	38.2	0.4 ± 0.0
9	Quercetin	40.1	1.0 ± 0.2
10	Kaempferol	47.8	3.4 ± 0.2
11	Apigenin	51.2	2.5 ± 1.6
12	Pinocembrin	55.6	20.4 ± 4.2
13	Chrysin	64.2	28.1 ± 0.6
14	CAPE	65.2	6.7 ± 1.0
15	Galangin	66.4	12.4 ± 3.0

^a^Values are the means ± SD (*n* = 3).
